# Epidemiology of Candidemia in Children over 7 Years in a Medical Center in Turkey

**DOI:** 10.1128/Spectrum.00453-21

**Published:** 2021-09-22

**Authors:** Dilek Yılmaz-Ciftdoğan, Ahu Kara-Aksay, Gülcan Erbaş, Ümit Başak Sarkış, Eda Karadağ-Oncel, Ayşe Berna Anıl, Maşallah Baran, Halil Er, Nisel Yılmaz

**Affiliations:** a Department of Pediatric Infectious Diseases, İzmir Katip Celebi University Faculty of Medicine, İzmir, Turkey; b Department of Pediatric Infectious Diseases, Health Sciences University, İzmir Tepecik Training and Research Hospital, İzmir, Turkey; c Department of Pediatrics, Health Sciences University, İzmir Tepecik Training and Research Hospital, İzmir, Turkey; d Department of Pediatric Intensive Care Unit, İzmir Katip Celebi University Faculty of Medicine, İzmir, Turkey; e Department of Pediatric Gastroenterology, İzmir Katip Celebi University Faculty of Medicine, İzmir, Turkey; f Department of Clinical Microbiology and Infectious Disease, Health Sciences University, İzmir Tepecik Training and Research Hospital, İzmir, Turkey; The Ohio State University

**Keywords:** candidemia, epidemiology, fluconazole resistance, nonparapsilosis, parapsilosis

## Abstract

The aims of the study were to describe *Candida* species in children with candidemia, to determine the changing epidemiology of candidemia over time in our tertiary care hospital, and to examine the demographic and clinical characteristics of patients with candidemia caused by parapsilosis and nonparapsilosis *Candida* spp. From 2012 to 2018, we identified a total of 126 cases of candidemia. The most commonly isolated *Candida* sp. was C. parapsilosis (*n* = 71, 56.3%), followed by C. albicans (*n* = 34, 26.9%). A total of 21 candidemia episodes (16.6%) were caused by other *Candida* species. Patients were divided into two groups (parapsilosis and nonparapsilosis) to identify any potential differences between the groups in terms of risk factors, mortality, and antifungal resistance. The median age of the patients, the median durations of the hospital and pediatric intensive care unit stay, receipt of immunosuppressive therapy within 2 weeks of developing candidemia, the rate of using total parenteral nutrition, need for mechanical ventilation, and receipt of carbapenems were statistically significantly higher in the parapsilosis group than in the nonparapsilosis group (*P* = 0.020, *P* = 0.001, *P* = 0.011, *P* = 0.036, *P* = 0.002, *P* = 0.038, and *P* = 0.004, respectively). The overall 30-day mortality rates (4.2% versus 3.6%) and resistance to fluconazole (33.8% versus 32.7%) were similar between the groups (*P* = 0.790 and *P* = 0.860, respectively). The distribution of *Candida* strains isolated in this study was consistent with the global trend, with C. parapsilosis being the most commonly identified species. Determining local epidemiologic data at regular intervals in candidemia cases is important in terms of determining both the changing epidemiology and empirical antifungal agents.

**IMPORTANCE** In our study, the changing epidemiology of *Candida* species in candidemia in children was evaluated. The dominance of Candida parapsilosis species in the changing epidemiology was remarkable. We found that fluconazole resistance was high in both parapsilosis and nonparapsilosis groups. Updating local epidemiologic data at certain intervals in candidemia cases is important in determining both the changing epidemiology and empirical antifungal agents.

## INTRODUCTION

Invasive candidiasis is an important cause of morbidity and mortality in hospitalized pediatric patients (1). Candida parapsilosis is second only to Candida albicans as a cause of systemic candidiasis. In recent years, the increase in the rate of C. parapsilosis candidemia is remarkable because of many risk factors, such as extreme prematurity, neutropenia or treatment with corticosteroids, or cytotoxic chemotherapy (2). Most patients with C. parapsilosis bloodstream infections (BSIs) are reported to have an underlying disease that requires the use of indwelling catheters and total parenteral nutrition (TPN) (3). Although the intestinal tract is shown as the main source of candidemia, it has recently emerged that skin colonization is also important in C. parapsilosis fungemia (4, 5). Accordingly, hand hygiene has an important role in preventing outbreaks of C. parapsilosis infections (6).

Different *Candida* species are associated with various degrees of tissue tropism, invasive potential, virulence, and antifungal susceptibility, which underlines the importance of center-specific surveillance studies for a better understanding of the optimal treatment and risk factors facilitating candidemia. The aim of this retrospective cohort study was to describe *Candida* species in children with candidemia and to determine the changing epidemiology of candidemia over time in our tertiary care hospital. In addition, it aimed to examine the clinical characteristics and risk factors of patients with candidemia, focusing on parapsilosis and nonparapsilosis *Candida* spp.

## RESULTS

During the 7-year study period, we identified a total of 126 cases of candidemia. The incidence of candidemia was 3.6 per 1,000 pediatric patient admissions. The incidence of candidemia was 8.5 per 1,000 pediatric intensive care unit (PICU) admissions. Specifically, the incidence rates of candidemia due to C. parapsilosis were 2.02 per 1,000 pediatric patient admissions and 4.8 per 1,000 PICU admissions. The distribution of C. parapsilosis and all other *Candida* species among patients with candidemia from 2012 to 2018 is shown in Table 1.

**TABLE 1:** Distribution of isolated *Candida* strains according to parapsilosis and nonparapsilosis group

Groups or species	Data from:							Total
	2012	2013	2014	2015	2016	2017	2018	
Parapsilosis group [*n* (%)]	4 (33.3)	8 (53.3)	15 (60)	13 (54.2)	13 (68.4)	9 (50)	9 (69.2)	71 (56.3)
Nonparapsilosis group [*n* (%)]	8 (66.7)	7 (46.7)	10 (40)	11 (45.8)	6 (31.6)	9 (50)	4 (30.8)	55 (43.7)
C. albicans	5 (41.7)	6 (40)	6 (24)	6 (25)	4 (21.2)	5 (27.8)	2 (15.4)	34 (26.9)
C. tropicalis	1 (8.3)	0	2 (8)	2 (8.2)	1 (5.7)	1 (5.5)	2 (15.4)	9 (7.1)
C. glabrata	1 (8.3)	1 (6.7)	1 (4)	1 (4.2)	1 (5.7)	2 (11.1)	0	7 (5.6)
C. lusitaniae	1 (8.3)	0	1 (4)	0	0	0	0	2 (1.6)
C. dubliniensis	0	0	0	1 (4.2)	0	0	0	1 (0.8)
C. guilliermondii	0	0	0	0	0	1 (5.5)	0	1 (0.8)
C. krusei	0	0	0	1 (4.2)	0	0	0	1 (0.8)
Total [*n* (%)]	12 (100)	15 (100)	25 (100)	24 (100)	19 (100)	18 (100)	13 (100)	126 (100)

The most commonly isolated *Candida* sp. was C. parapsilosis (*n* = 71, 56.3%), followed by C. albicans (*n* = 34, 26.9%). A total of 21 candidemia episodes (16.6%) were caused by other *Candida* species (7.1% C. tropicalis, 5.6% C. glabrata, 1.6% C. lusitaniae, 0.8% C. krusei, 0.8% C. dubliniensis, and 0.8% C. guilliermondii) (Table 1).

Risk factors for candidemia, including patient characteristics, comorbidities, clinical procedures, and medications, between the parapsilosis group and nonparapsilosis group are shown in Table 2. The median age of the patients was higher in the parapsilosis group than in the nonparapsilosis group (*P* = 0.02). The median durations of the hospital and PICU stay were higher in parapsilosis group than in the nonparapsilosis group (*P* = 0.001, *P* = 0.011, respectively). The parapsilosis group had higher rates of receipt of immunosuppressive therapy within 2 weeks prior to candidemia than the nonparapsilosis group (*P* = 0.036). Also, higher rates of use of TPN (*P* = 0.002), need for mechanical ventilation support (*P* = 0.038), and receipt of carbapenems (0.004) were detected in the parapsilosis group than in the nonparapsilosis group. There were no statistically significant differences detected between the two groups for the rates of fluconazole use within 2 months prior to candidemia (*P* = 0.76).

**TABLE 2:** Risk factors for the parapsilosis and nonparapsilosis groups

Factor	Parapsilosis group (*n* = 71) [*n* (%)]	Nonparapsilosis group (*n* = 55) [*n* (%)]	*P* value
Age (mo)^*a*^	23 (3–192)	12 (4–186)	**0.020** ^*c*^
Sex (male/female)	40/31	28/27	0.540
Length of hospital stay^*a*^	52 (9–255)	21 (7–147)	**0.001** ^*c*^
Length of PICU stay^*a*^	15 (3–235)	3 (2–201)	**0.011** ^*c*^
Comorbidities and clinical procedures^*b*^			
* *Malignancy	1 (1.4)	2 (3.6)	0.822
* *Prior solid organ transplantation	10 (14.1)	5 (9.1)	0.561
* *Prior hematopoetic stem cell transplantation	1 (1.4)	1 (1.8)	0.850
* *Receipt of immunosuppressive therapy within 2 wks prior to candidemia	20 (28.2)	7 (12.7)	**0.036** ^*c*^
* *Surgery of the gastrointestinal tract within 2 wks prior to candidemia	21 (29.6)	14 (25.5)	0.750
* *Neutropenia	2 (2.8)	1 (1.8)	0.715
* *Need for mechanical ventilation support	35 (49.3)	17 (30.9)	**0.038** ^*c*^
* *Renal failure	1 (1.4)	1 (1.8)	0.850
* *Peritoneal dialysis	3 (4.2)	2 (3.6)	0.860
* *Hemodialysis	2 (2.8)	1 (1.8)	0.715
* *Use of total parenteral nutrition	49 (69)	22 (40)	**0.002** ^*c*^
* *Presence of central venous catheter	53 (74.6)	36 (65.5)	0.350
* *Presence of urinary catheter	34 (47.9)	32 (58.2)	0.330
* *Presence of temporary nasogastric tube	23 (32.9)	19 (34.5)	0.840
* *Presence of permanent nasogastric tube	5 (7)	4 (7.3)	0.960
* *Antibiotic use within 2 wks prior to candidemia			
* *Carbapenemes	60 (84.5)	33 (60)	**0.004** ^*c*^
* *Expanded spectrum cephalosporins	40 (57.1)	32 (58.1)	0.820
* *Aminoglycosides	39 (54.9)	30 (54.5)	0.780
* *Vancomycin	27 (38.02)	23 (41.8)	0.850
* *Teicoplanin	33 (47.1)	18 (32.7)	0.140
Fluconazole use within 2 mo prior to candidemia^*b*^	9 (12.7)	6 (10.9)	0.760
Overall 30-day mortality rate^*b*^	3 (4.22)	2 (3.63)	0.860

a Values were given as median (min-max).

b Values were given as a percentage.

c The *P* value <0.05 is shown in bold.

The antifungal susceptibility of *Candida* species in both groups is shown in Table 3. A total of 24 isolated C. parapsilosis strains (33.8%) were resistant to fluconazole, and 18 of the other isolated *Candida* species strains (32.7%) were resistant to fluconazole. In both groups, intermediate resistance was detected in 2 isolated *Candida* strains. There was no statistically significant difference in the rates of resistance and intermediate resistance to fluconazole between the two groups (*P* = 0.790 and *P* = 0.890, respectively). Itraconazole resistance was higher among the nonparapsilosis group than the parapsilosis group (*P* = 0.001). Voriconazole resistance rates were similar between the groups (*P* = 0.490), while intermediate resistance rates were higher among the parapsilosis group than the nonparapsilosis group (*P* = 0.010). No resistance or intermediate resistance to amphotericin B was observed in the parapsilosis group. In the nonparapsilosis group, 2 isolates (C. krusei, C. guilliermondii) were found to be amphotericin B resistant. There were no statistically significant differences detected between the two groups for the rates of flucytosine resistance and intermediate resistance (*P* = 0.18 and *P* = 0.43, respectively).

**TABLE 3:** Antifungal susceptibility testing and antifungal-resistant candida strains

*Candida* species	Antifungal susceptibility^*a*^ of (*n*):														
	Fluconazole			Itraconazole			Voriconazole			Amphotericin B			Flucytosine		
	S	I	R	S	I	R	S	I	R	S	I	R	S	I	R
C. parapsilosis (*n* = 71)	45	2	24	54	15	2	52	8	11	71	0	0	71	0	0
All other *Candida* spp. (*n* = 55)	35	2	18	32	9	14	43	0	12	53	0	2	52	2	1
C. albicans	22	1	11	21	5	8	26	0	8	34	0	0	34	0	0
C. tropicalis	5	0	4	4	1	4	5	0	4	9	0	0	9	0	0
C. glabrata	4	0	3	4	1	2	7	0	0	7	0	0	7	0	0
C. dubliniensis	1	0	0	1	0	0	1	0	0	1	0	0	1	0	0
C. krusei	0	1	0	0	1	0	1	0	0	0	0	1	0	1	0
C. guilliermondii	1	0	0	0	1	0	1	0	0	0	0	1	1	0	0
C. lusitaniae	2	0	0	2	0	0	2	0	0	2	0	0	0	1	1
Total (*n* = 126)	80	4	42	86	24	16	95	8	23	124	0	2	123	1	2

a S, susceptible; I, intermediate; R. resistant.

The fluconazole resistance was determined in 4 (44.4%) patient isolates among 9 patients who had fluconazole use within 2 months prior to candidemia with C. parapsilosis compared to 2 (33.3%) patient isolates among 6 patients with other *Candida* species.

The overall 30-day mortality rate in candidemia episodes was 3.9% (5 out of 126 patients). The overall 30-day mortality rate for the parapsilosis group was 4.2% (3 out of 71 patients) compared to a rate of 3.6% for the nonparapsilosis group; the difference was not statistically significant (*P* = 0.860).

Logistic regression analysis was performed for risk factors that showed a statistically significant difference between the parapsilosis and nonparapsilosis group. The receipt of immunosuppressive therapy within 2 weeks prior to candidemia was found to be associated with a 3.44-fold increase in the developing candidemia due to C. parapsilosis (95% confidence interval [CI], 1.25 to 10; *P* = 0.018) and need for mechanical ventilation support, with a 2.56-fold increase for developing candidemia due to *C. parapsilosis* (95% CI, 1.16 to 5.88; *P* = 0.021).

## DISCUSSION

*Candida* spp. are the third most common etiology of health care-associated BSIs (1, 3). Identification of risk factors for invasive candidiasis is especially important in the development of preventive strategies. Overall, it is known that the risk of candidemia increases as the length of stay in the hospital, especially in the intensive care unit (ICU), and invasive interventions such as the insertion of central venous catheter (CVCs) and the use of TPN increase (7, 8). In our study, in accordance with the literature, patients had a high rate of TPN (56.3%) and CVC use (70.6%) in both groups.

The distribution of *Candida* species shows regional differences (9). In our study, C. parapsilosis (56.3%) was the most frequently isolated species, followed by C. albicans (26.9%), C. tropicalis (7.1%), C. glabrata (5.6%), C. lusitaniae (1.6%), C. krusei (0.8%), C. dubliniensis (0.8%), and C. guilliermondii (0.8%). In a study performed on 102 children with nosocomial candidemia in our country within a 9-year period, Celebi et al. (10) reported that the three most common causes of candidemia were C. albicans (39.2%), C. parapsilosis (21.6%), and C. tropicalis (15.7%). A study conducted from 2004 to 2012 showed that C. albicans was the most commonly identified species from candidemia episodes (11). In another study performed on children with malignancy and nosocomial candidemia in a similar region, nonalbicans candidemia was determined in 81.4% of 135 candidemia episodes during the study period (12). In a recent study from Turkey (13), the distribution of *Candida* strains was consistent with our results, showing that C. parapsilosis was the most common strain in children.

C. parapsilosis was first isolated as an agent in endocarditis developing in an intravenous (i.v.) drug-dependent individual in 1940 (14). Its incidence has increased greatly in the last 30 years (15). Various factors, such as affinity for intravascular catheters and prosthetic materials, an increase of TPN use, and transmission from the colonized hands of health care workers, have been shown to cause this increase in C. parapsilosis infection (4). In a surveillance study in Barcelona, Spain, Almirante et al. (8) revealed many risk factors for C. parapsilosis BSI, including vascular catheterization, previous antibiotic use, prior immunosuppressive therapy, malignancy, transplant receipt, neutropenia, and previous colonization. In our study, prolonged stay in the hospital and PICU, receipt of carbapenems and immunosuppressive therapy within 2 weeks of developing candidemia, the need for mechanical ventilation support, and the use of TPN were greater risk factors for fungemia in the parapsilosis group than in the other group.

Independent risk factors associated with the development of candidemia due to C. parapsilosis determined through logistic regression analysis were receipt of immunosuppressive therapy within 2 weeks of developing candidemia (odds ratio [OR], 3.44; 95% CI, 1.25 to 10) and the need for mechanical ventilation support (OR, 2.56; 95% CI, 1.16 to 5.88). As is known, among *Candida* species, C. parapsilosis is the second most common biofilm producer after C. albicans. Compared with C. albicans, C. parapsilosis forms less complex and thin biofilms (16). The normal gastrointestinal barrier serves an important function in preventing invasive *Candida* infections. The destruction of this flora in major surgery is a potential risk factor for candidiasis (16). Additionally, C. parapsilosis also grows rapidly in TPN administered to patients without oral intake, especially patients in the ICU and those with gastrointestinal system disorders (16).

The ease of formation of C. parapsilosis biofilms in the presence of high-glucose or lipid-rich media is associated with the increased incidence of C. parapsilosis-induced candidemia in patients receiving parenteral nutrition (17, 18). All these results show that patients who require medical and/or nutritional support are the most likely to develop invasive *Candida* infections, especially when their hospital stay is prolonged. Neonates were not included in this study because their risk factors and epidemiologic characteristics for invasive fungal infections are very different from those of older children. It has been reported in recent studies that risk factors such as low birth weight and prematurity are important for C. parapsilosis in neonates (5).

Unlike the prior-colonization-dependent vertical transition of C. albicans, C. parapsilosis transitions horizontally; therefore, C. parapsilosis is one of the most frequently isolated *Candida* species in ICUs (19). The ability of this pathogen to form biofilms on permanent devices and its easy transmission through colonizing the hands of health care workers has been associated with the occurrence of hospital outbreaks and high mortality rates (20–22). These data show that hand hygiene is very important in the formation of C. parapsilosis infections. In our study, it was observed that C. parapsilosis infections did not cluster together, so there was no outbreak.

It is known that there is azole resistance in C. parapsilosis. In our study, during the 7-year study period, out of 71 C. parapsilosis strains, 33.8% were resistant to fluconazole. However, 18 of the other isolated *Candida* species (32.7%) were also resistant to fluconazole. There was no statistically significant difference between the groups regarding fluconazole resistance rates. Itraconazole resistance was higher among the nonparapsilosis group than in the parapsilosis group. Voriconazole resistance rates were similar between the groups. Thomaz et al. (23) revealed that eight (57.1%) of 14 C. parapsilosis isolates were fluconazole-resistant. It is well known that azole resistance can develop with previous or current use of fluconazole (24–27) and even with the use of systemic antibiotics (28–30). All of our patients had a history of antibiotic use within 2 weeks of developing candidemia. We determined that carbapenems or expanded-spectrum cephalosporins, in particular, were used in almost all patients. However, in our study, we found that the rate of fluconazole use within 2 months of developing candidemia was lower, contrary to the literature (7, 27).

In our study, the overall 30-day mortality rate in candidemia episodes was 3.9%. Belet et al. (7) showed that 22.8% of patients died in the first 30 days. In another study, Pappas et al. (1) also revealed that C. albicans was associated with high overall mortality among children (29%). In a previous study, the overall 30-day mortality rate in candidemia episodes was 23.4% (13). Karadag-Oncel et al. (11) showed that the C. albicans-related mortality in their study was 34.1% compared with a mortality rate of 23% for C. parapsilosis. These findings differ from our results; we had a low mortality rate in both the parapsilosis and nonparapsilosis groups (4.2% versus 3.6%). We associated this significant difference in mortality rates with the fact that only 38.8% of the patients had comorbid diseases and only 2.3% were neutropenic in our study.

A study conducted in Spain showed that the mortality rate was lower in patients with C. parapsilosis than in those with C. albicans infection, as in our study (31). In another pediatric study, Kollef et al. (32) found that C. parapsilosis had the highest mortality rate of all *Candida* species. Overall, pediatric studies have shown that candidemia has a high mortality rate. Studies demonstrated that mortality was closely related to both the timing of treatment and resource control (33–38). This means that early intervention with appropriate antifungal therapy and removal of a contaminated CVC are generally associated with better overall outcomes. Therefore, a fungal etiology should be considered in appropriate clinical settings, especially in patients with risk factors. Empirical antifungal therapy is required in these patients because early diagnosis and treatment are associated with reduced mortality.

To the best of our knowledge, ours is the largest pediatric study comparing BSIs due to C. parapsilosis and nonparapsilosis *Candida* species; however, it has some potential limitations. The first is that it was a single-center study, and the second is its retrospective design. Future prospective multicenter studies with larger patient numbers will further contribute to the literature.

The changing epidemiology of *Candida* species in candidemia in children was evaluated in our study. The dominance of C. parapsilosis species in the changing epidemiology was remarkable. We found that fluconazole resistance was high in both parapsilosis and nonparapsilosis groups. Updating local epidemiologic data at certain intervals in candidemia cases is important in determining both the changing epidemiology and empirical antifungal agents.

## MATERIALS AND METHODS

### Study design, setting, and patients.

We conducted this retrospective cohort study at İzmir Tepecik Training and Research Hospital, a university-affiliated referral medical center. From 1 January 2012 to 31 December 2018, all the hospitalized pediatric patients (18 years of age and younger) who had diagnosed candidemia were reviewed. Neonates were not included in the study.

Demonstration of growth of *Candida* spp. in blood culture was defined as candidemia. Patients who had diagnosed candidemia were identified using the records of the hospital and mycology laboratory.

In patients with multiple episodes of candidemia during the study period, the first episode was evaluated in this study. During the study period, the patients who had developed candidemia due to C. parapsilosis were referred to as the “parapsilosis group,” while the patients who had developed candidemia with different *Candida* spp. other than C. parapsilosis were referred to as the “nonparapsilosis group.” Also, the 30-day mortality, within 30 days from the onset of candidemia, was evaluated. The study was approved by the institutional review board of the İzmir Tepecik Training and Research Hospital.

### Data collection.

The clinical and laboratory data were collected from the medical records. The data obtained included age, sex, duration of hospitalization prior to infection, history of hospitalization in the pediatric intensive care unit (PICU), duration of hospitalization in the PICU, comorbid conditions, prior use of antimicrobial medications, and presence of a medical device.

Comorbid conditions included malignancy, prior solid organ transplantation, prior hematopoietic stem cell transplantation, neutropenia (absolute neutrophil count of <500 mm^3^), renal failure, use of TPN, need for mechanical ventilation support, receipt of immunosuppressive therapy and use of antimicrobial medications within 2 weeks prior to candidemia, and receipt of abdominal surgical procedure within 2 weeks prior to candidemia. The use of antimicrobial medications within 2 weeks prior to the diagnosis of candidemia were recorded. In addition, fluconazole use within 2 months prior to candidemia was also considered. The presence of a central venous catheter (CVC), percutaneous endoscopic gastrostomy, urinary catheter, peritoneal dialysis catheter, and hemodialysis catheter was also recorded.

### Identification of *Candida* species and antifungal susceptibility testing.

Blood samples were taken under sterile conditions from patients with suspected candidemia. The samples sent to the microbiology laboratory were placed in BacT/Alert medium bottles and incubated in a BacT/Alert 3D system (BioMérieux, France) for 7 days. Gram staining was performed from the samples which gave a positive growth signal during incubation. Samples found as yeast at the end of the Gram staining were inoculated on Sabouraud dextrose agar (SDA) and 5% sheep blood agar plates. At the end of the incubation period, the germ tube test, Tween 80 agar inoculation, CHROMagar inoculation, and identification with the API ID 32C (BioMérieux, France) were conducted on the colonies. Duplicate isolates from the same patients were excluded.

One to two colonies which grew on the plates were suspended in saline (NaCl, 0.85%), and the turbidity was adjusted to 0.5 McFarland standard. RPMI 1640 medium supplemented with 2% glucose and with the pH adjusted to 7.0 and morpholinepropanesulfonic acid (MOPS) buffer were used for susceptibility tests. Yeast suspension was evenly spread onto the surface of the medium. Petri plates were allowed to dry for 10 to 15 min before the Etest (BioMérieux, France) strips were applied. The Etest procedure was performed according to the manufacturer’s directions using fluconazole, voriconazole, itraconazole, posaconazole, amphotericin B, and anidulafungin test strips. MIC values were recorded after 24 to 48 h of incubation at 35°C. Fluconazole, voriconazole, itraconazole, anidulafungin, and flucytosine were evaluated according to CLSI breakpoint values, while posaconazole and amphotericin B were evaluated with EUCAST breakpoint values (39, 40). The results were evaluated as ≤8 μg/ml susceptible (S), 16 to 32 μg/ml intermediate (I), and ≥64 μg/ml resistant (R) for fluconazole; ≤1 μg/ml S, 2 μg/ml I, and ≥4 μg/ml R for voriconazole; ≤0.125 μg/ml S, 0.25 to 0.5 μg/ml I, and ≥1 μg/ml R for itraconazole; ≤2 μg/ml S and ≥2 μg/ml R for anidulafungin; ≤1 μg/ml S and ≥1 R for amphotericin B; and ≤0.064 S and ≥0.064 R for posaconazole (39, 40). Routine quality control of antifungal susceptibility tests was performed with ATCC 22019 C. parapsilosis and ATCC 6258 C. krusei strains.

### Statistical analysis.

Data were analyzed with SPSS software version 23.0. Continuous variables were summarized using the median and interquartile range (IQR), while categorical variables were summarized using frequencies and percentages. Categorical variables were compared with the Chi-square test or Fisher’s exact test. A *P* value of <0.05 was considered significant. Multivariate analysis using logistic regression was performed to identify associations between variables and risk factors for developing candidemia due to C. parapsilosis. All variables with a univariate *P* value of <0.20 were considered for inclusion in the multivariate model.
